# Birth cohort studies using symptom diaries for assessing respiratory diseases–a scoping review

**DOI:** 10.1371/journal.pone.0263559

**Published:** 2022-02-10

**Authors:** Susan Langer, Bianca Klee, Cornelia Gottschick, Rafael Mikolajczyk

**Affiliations:** Institute for Medical Epidemiology, Biometrics and Informatics, Interdisciplinary Center for Health Sciences, Medical School of the Martin-Luther University Halle-Wittenberg, Halle, Germany; UKBB Universitats-Kinderspital, SWITZERLAND

## Abstract

**Background:**

Respiratory infections are the most frequent health problem in childhood leading to morbidity and socioeconomic burden. Studying symptoms of respiratory infections in home based settings requires dedicated prospective cohort studies using diaries. However, no information is available on which birth cohort studies using symptom diary data. A review of birth cohort studies with available symptom diary data, follow-up data, and bio samples is needed to support research collaborations and create potential synergies.

**Methods:**

We conducted a scoping review of birth cohort studies using diaries for the collection of respiratory symptoms. The scoping review was conducted in accordance with the PRISMA Extension. We searched the electronic databases PubMed, Embase, Web of science and CINAHL (last search November 2020) resulting in 5872 records (based on title and abstract screening) eligible for further screening.

**Results:**

We examined 735 records as full text articles and finally included 57 according to predefined inclusion criteria. We identified 22 birth cohort studies that collect(ed) data on respiratory symptoms using a symptom diary starting at birth. Numbers of participants ranged from 129 to 8677. Eight studies collected symptom diary information only for the first year of life, nine for the first two years or less and six between three and six years. Most of the cohorts collected biosamples (n = 18) and information on environmental exposures (n = 19).

**Conclusion:**

Information on respiratory symptoms with daily resolution was collected in several birth cohorts, often including related biosamples, and these data and samples can be used to study full spectrum of infections, particularly including those which did not require medical treatment.

## Introduction

Acute respiratory infections (ARI) are the most common health problems in early childhood [1], and the most common causes of death in children under the age of 5 years worldwide [2]. Although most of ARI are not severe [1], they are frequent and cause a high number of doctor visits [3, 4], hospitalizations, antibiotic prescriptions [3, 5], a high socioeconomic burden [6, 7], and absenteeism in education and work [8]. Infections with respiratory viruses (e.g. human rhinovirus, enterovirus and adenovirus) in early childhood may influence the development of chronic and immune-mediated diseases such as asthma, type II diabetes and obesity later in life [9, 10], however, it is not clear how. Research in this area therefore remains very important. About half of ARI in infancy do not lead to consultation of a physician, which leads to a significant underestimation of the true incidence [7, 11–14].

For many specific etiologic questions, it is necessary to have information on a light course of infections. However, this requires prospective data, and collecting this information provides a large challenge. Therefore, the accurate recording of ARI during early childhood needs thoroughly planned prospective birth cohort studies using symptom diaries. Symptom diaries are tools for participants to record symptoms daily in a systematic manner over a defined period [15]. This allows analyses of a prospective evolution of symptoms linked to specific pathogens (if biosamples allow identification of pathogens) and accounting for previous infection episodes, age at infection and time since the last episode. Symptom diaries have a long tradition in evaluating acute infections [13, 16], but are also very demanding. Therefore, other approaches such as retrospective questionnaires, reports from clinical visits or retrospective interviews are more often used [17–23], but have to accept the possibility of recall bias [24]. Additionally, retrospective assessment does not allow studying the duration of symptoms or the change of symptoms over time adequately.

In birth cohort studies, newborns or infants are recruited before, at, or shortly after birth and observed over many years to examine associations between early life exposures and outcomes later in life [25]. Many birth cohorts have been focusing on respiratory infectious diseases and various aspects of these studies were covered in systematic and scoping reviews [26–32].

However, none of them focused on the prospective recording of respiratory infections using symptom diaries and the combination with biosamples. This information can be used to study patterns and severity of symptoms associated with pathogens and other factors such as susceptibility, immune system development or the development of chronic diseases later in life.

In this scoping review, we aimed to compile, map, and compare the existing birth cohort studies collecting symptom diary information on respiratory infections in childhood starting at birth to promote potential research collaborations and exploit synergies. We considered respiratory infections, the collection of biosamples and environmental exposures in order to identify studies suitable for providing a holistic understanding of the association of ARI as exposure with potential long-term sequelae in a life course perspective.

## Material and methods

### Inclusion and exclusion criteria

This scoping review was performed using PRISMA Extension [33]. We included (1) prospective observational birth cohort studies that (2) examined healthy newborns and (3) used a symptom diary related to respiratory infections. We defined a birth cohort study as a study that recruits children prospectively prior to birth or up to the age of four months.

We excluded all other study designs such as randomized controlled trials, case control studies, cross sectional, qualitative studies and publications in languages other than English. In addition, we excluded studies that focused only on children with chronic lung disease, studies that included children only when a specific symptom or virus was identified, studies that recruited children older than 4 months, or studies that focused only on preterm infants. There were no requirements for the duration of the symptom diary or a time limit for the date of publication.

### Search strategy

In a first step, we searched four electronic databases (last search 11.2020): MEDLINE; EMBASE; CINAHL; Web of Science. The search strategy was developed to include several terms referring to respiratory infections in children, symptom diary, and study design. In Table 1, we present the search strategy that was used for MEDLINE and was applied with minor changes to the other databases.

**Table 1:** Search strategy for PubMed.

**(Child [TIAB] OR Children [TIAB] OR Childhood [TIAB] OR newborn [TIAB] OR infant [TIAB] OR newborn [MH] OR infant [MH] OR child [MH])**
**AND**
(“birth-cohort” [TIAB] OR birth* [TIAB] OR “Prospective-study” [TIAB] OR “longitudinal study” [TIAB] OR “follow-up study”[TIAB] OR cohort* [TIAB] OR “Birth cohort study” [TIAB]OR “cohort studies”[MH])
**AND**
**(“respiratory-tract-infection” [TIAB] OR“Infectious diseases” [TIAB] OR “Infection diseases” [TIAB] OR “communicable diseases” [TIAB] OR “respiratory infections” [TIAB] OR “virology” [TIAB] OR “immunology” [TIAB] OR “virus disease” [TIAB] OR “viral disease” [TIAB] OR “infectious” [TIAB] OR “bacterial infection” [TIAB] OR “infections” [TIAB] “respiratory tract infections"[MH]"communicable diseases"[MH] OR “virology"[MH] OR “allergy and immunology"[MH] OR" virus diseases"[MH] OR "bacterial infections"[MH] OR "infection"[MH])**
**AND**
**(“Daily-diary-study” [TIAB] OR Diary [TIAB] OR “daily-diary” [TIAB] OR “daily-diaries” [TIAB] OR “daily-observation” [TIAB] OR “daily- records” [TIAB] OR “self-reported-Questionnaire”[TIAB] OR “Symptom records”[TIAB] OR “Symptom questionnaire”[TIAB]OR “Symptom diary” [TIAB] OR “Symptom diaries” [TIAB])**

Cohort identification took place between January 2019 and November 2020.

One researcher (SL) screened titles and abstracts of identified studies with respect to the inclusion and exclusion criteria. In a second step, two authors (SL and BK) conducted a backward search whereby bibliographies of the included studies were screened to identify any studies that might have been missed in the first screening. Subsequently, titles, abstracts and full text articles of additionally identified references were screened according to the inclusion and exclusion criteria. The results of both screeners were compared and discrepancies were discussed. In a third step, we scanned other sources such as registers of birth cohort studies (BirthCohorts.net (https://www.birthcohorts.net/) (11.2020), Asthma Birth Cohorts Database (https://asthmabirthcohorts.niaid.nih.gov/) and performed a grey-literature search and reviewed other reviews of birth cohorts (last search 11.2020). The final search results were exported into EndNote and duplicates were removed. Two authors (SL and BK) extracted data from all included birth cohorts independently, based on predefined criteria, compared the results, and discussed disparities. Data of interest included: cohort name, country in which the study took place, number of participants, enrollment period, follow-up times without symptom diary, symptom diary duration (from birth), at what age questionnaires were answered, if and at what time communication was via email, telephone and if interviews were conducted, if and when home visits, examinations in the clinic or in general took place. In addition, we extracted data on what biosamples were collected and at what time. In addition, it was of interest which risk factors for respiratory infections were of interest in each study. In only three of the cohorts a cohort profile was available. Thus, information on the study design was often taken from several publications focusing on different aspects of the same cohort study. It was often not possible to extract complete data from these specialized publications. Therefore, the last step was to validate and complement the extracted results by contacting the authors of the most recent publications of the respective birth cohorts via email and asking them to confirm or complete the available information (February-November 2020). Reminders were sent 3–5 weeks later. Investigators of 14 out of 22 studies (63.6%) responded to our inquiries.

## Results

### Search results

**Fig 1:**
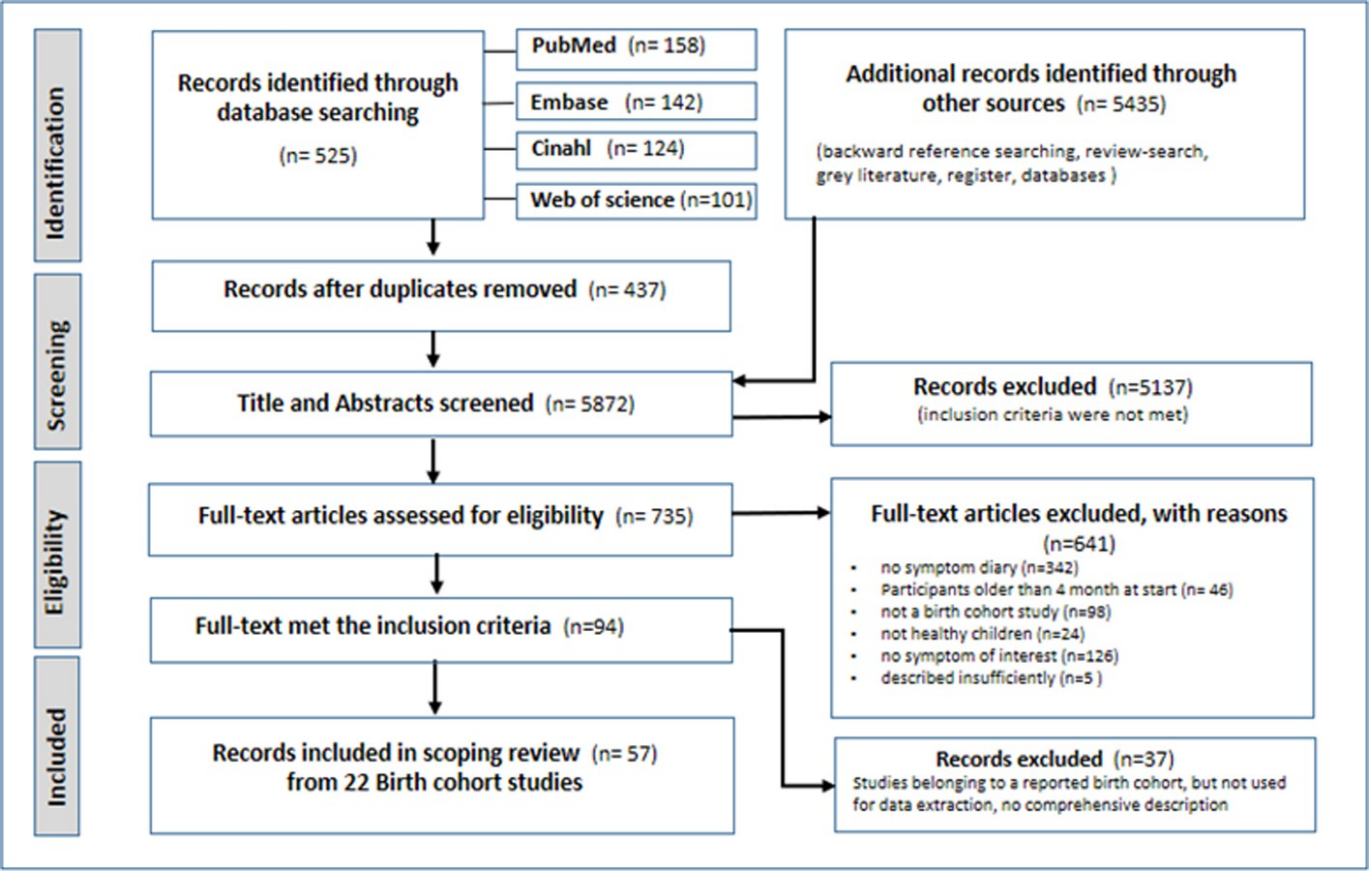
PRISMA-flow diagram.

We identified 5960 records by database search and by backward reference, register and review search (Fig 1).

After removing duplicates, 5872 records were included in the screening of abstracts and titles of which 735 were found eligible for full-text analysis based on our inclusion criteria. Of these, a final number of 57 full-text articles met the inclusion criteria. Based on the articles, we identified 22 birth cohort studies that collect data or have already completed data collection on respiratory symptoms using a symptom diary (Table 2).

**Table 2:** Study characteristics sorted by descending enrollment period of birth cohort studies with approximately N = 26000 participants.

	REF	COUN-TRY	PARTICIPANTS (newborns)	ENROLLMENT PERIOD	SYMPTOM DIARY DURATION (from birth)	FOLLOW UP (FU) (without symptom diary)	QUESTIONNAIRE	CALLS/ INTERVIEW/ EMAIL	HOME-VISITS	CLINICAL VISITS/EXAMINATIONS
PittsburghCohort(*)†	[42]	USA	n = 244	07/1985-04/1886	until 3 yrs	every 2 wks	-	every 2 wks	-	-
Adelaide-Cohort (*)†	[38]	South AUS	n = 1981	05/1987 04/1988	until 2 yrs	after birth, once every 3 m until 24 m	9 and 18 m	after delivery and discharge, then every 3 m until 2 yrs	-	-
New Mexico-Cohort(*)†	[11, 16, 36]	USA	n = 1205 ***χ*** [17]	01/ 1988 06/1990 ***χ***	until 18 m	every 2 wks calls/ Interview	-	every 2 wks, up to age 18 m	at enrolment	-
Raine Study *	[43–46]	West AUS	n = 2868 ***χ*** [46]	05/1989-11/1991 ***χ*** (53)	first y of life	18, 34 wks gestation, birth, 1,2,3,5,8,10,14, 17,18,20,22,27 yrs	at age of 1, 2, 3, 4, 5, 8, 10, 14,17, 20 and 22, 27 yrs	telephone FU/ interviews ϕ	-	at age of 1, 2, 3, 5, 8, 10, 14, 17, 18, 20, 22 yrs
Perth-Cohort (*)†	[5, 47]	AUS	n = 263	07/1996-06/1998	until 5 yrs	birth, 6 wks, 6, 12 m of age, then yl	not described in detail	telephone calls until resolution of the child’s symptoms	home visit for respiratory symptoms	at 6 wks, 6 and 12 m of age, and then yl
VIGALL (*)	[34]	NLD	n = 126	08/1996-11/1998	wkly symptom card until 2 yrs	birth, 3m, 6m, 12m, 18m, 24m	birth, 3, 12, 24 m	-	during signs of URI	6 m, 12 m, 18 m, 24 m
PEIC (*)‡	[41, 48]	CAN	n = 332	06/1997 1999	until 2 yrs	twice mtly until 2 yrs	-	phoned twice mtly	-	-
Allergy-flora (*)	[49, 50]	SWE	n = 187	1998 2003	first y of life	6, 12 m until 18 m Contact to parents at wk 1, 2, 4 and 8 until 2005	-	at enrolment, then 6 and 12 m	-	at 18 m of age and if allergic symptoms occur
COPSAC 2000 (*)	[51, 52]	DK	n = 411	08/1998 12/2001	until 6 yrs ϕ	2, 4 wks ϕ, every 6 m until the age of 7 yrs, then at the age of 9, 12, 18 yrs	-	at enrolment, 1, 6, 12 m	-	at the age of 2 wks, every 6 m until the age of 7; if symptoms occur
WHIST-LER *	[53–57]	NLD	n = 2133 [58]	12/2001-01/2013	first y of life	at birth, mtly during the first 12 m, 5, 8 yrs	birth, mtly during first y, then yl	as reminder	to collect dust	2^nd^ or 3^rd^ wk of life /FU 5 and 8 yrs
PASTURE (*)	[58–60]	AUT DEU FIN FRA CHE	n = 1133	08/2002 03/2005	2 m until 1 y	before birth 2, 12, 18, 24, 36, 48, 60, 72 m, at the age of 6 yrs, 10,5 yrs (until 2015)	before birth, 2, 12, 18, 24 m, and then yl until age of 6, 10,5 yrs	before birth, 2, 12, 18, 24 m, and then yl until age of 6 yrs	age of 2 m	age of 1y
Kopen-hagen-Cohort(*)†	[37, 61, 62]	DK	n = 242 ***χ***	05/2004 05/2005	first y of life	mtly until 1 y (1 May 2006)	-	at the first home visit, repeated every second m	mtly	mtly home visits
TEDDY Study *	[35, 63, 64]	USA/EUR	n = 8677	12/2004-02/2010	3 m until 2 yrs, then appropriate book	every 3 m until 4 yrs, then yl until the age of 15 yrs	3, 9 m, up-dated after 2 yrs, then every 4 y	during visits biannually at 4 yrs of age	-	every 3 m until the age of 4yrs, then biannual, subgroup every 3 m
**Madigan** Childcare Study (*) **‡**	[65–67]	USA	n = 225 ¥	02/2006-04/2008 10/2008-06/2009	STA was completed by the child’s parent for 10 days following illness onset for 2 y	interviews until 40 m of age ***χ***	-	at enrolment and if symptoms occurred	at illness onset	a study physician documented visit/ nurse contact in childcare site
Utrecht-Cohort (*)†	[68–71]	NLD	n = 291 ***χ*** (39)	03/2006-02/2010***χ***	first y of life	1 and 12 m	at 1m and 1 y of age	around birth, 3 wks after delivery	first respi-ratory infection	to withdraw blood at first m
STEPS *	[72–75]	FIN	n = 1827	01/2008-04/2010	first two yrs of life daily, then wkly until 5 yrs of age	before and after birth, 13, 18, 24 m. until the age of 2 yrs	before birth, birth, 13, 18, 24 m, then yl	no	no	if symptoms occur/ age of 2, 13, and 24 m.
COPSAC 2010 *	[76]	DK	n = 700	2008/2010	until 3 yrs ϕ	24, 36 wks gestation,1 wk, 1, 3, 6 m, then every 6 m until 36 m, then yl until 6 yrs, then at the age of 8, 10 yrs	online survey from 2016 onwards ϕ	at regular intervals between visits	-	week 24 of gestation, 1, 3, 6 m, then every 6 m until 36 m, then yl until 7yrs; if symptoms occur
**DIABIMMUNE**	[77–80]	FIN EST RUS	n = 563	09/2008–10/2013	3m until 3 yrs	3, 6, 12, 18, 24, 36 m	3, 6, 12, 18, 24, 36 m	**-**	-	3, 6, 12, 18, 24, 36-m
OrChid (*)	[12, 81–83]	AUS	n = 158	09/2010-10/2014	until 2 yrs	every 3 m until 2nd birthday	no	telephone-interview 3‐mtly	no	after delivery
NPICS (*)	[39, 84]	NIC	n = 518 n = 1705 ¥	2011–2013	until 2 yrs (subgroup)	until 2 yrs, wkly in diary subgroup	at enrollment, then yl.	yl calls and email as reminder	for part who did not attend FU	yl/ first signs of influenza-like illness
PATCH (*)	[85, 86]	TWN	n = 387	01/2012-11/2014	first y of life	1, 2, 4, 6, 12 m	at each planned visit	calls, interviews, emails	-	regular visits at clinic and examinations, if symptoms occur
Loewen KIDS *	[87]	DEU	n = 782	11/2014-02/2018	until 6 yrs	at birth, 3,6,12,18, 24 m, then yl until age of 15 y	at birth, 6 m, age 1 till 15 yl.	email as reminder	no	to withdraw blood
**wk** **wkly** **m** **mtly** **y** **yl** **part**	**week** **weekly** **months** **monthly** **year** **yearly** **participant**		**¥** (*) * - ***χ*** **†**	**over all sample size** **complete** **active** **data not reported** **varies according to publication** **if no study name was known the location of the primary centre is given**	**‡** **‡ Madigan** **Childcare-Study** **‡PEIC** **URI** **ϕ** **-**	**abbreviated cohort name** **Madigan Army Medical Centre-Childcare Study** **Prince Eduard Island Cohort** **upper respiratory infection** **validated through study stuff- different to data in publication** **Data not reported**				

### Characteristics of included cohorts

The majority of included birth cohorts were conducted in Europe (n = 12), four were conducted in Australia, four in the United States of America and one each in Canada, Nicaragua and in Taiwan. Three of those were conducted in several countries, such as the TEDDY Study, which was conducted in the United States and Europe.

**Fig 2:**
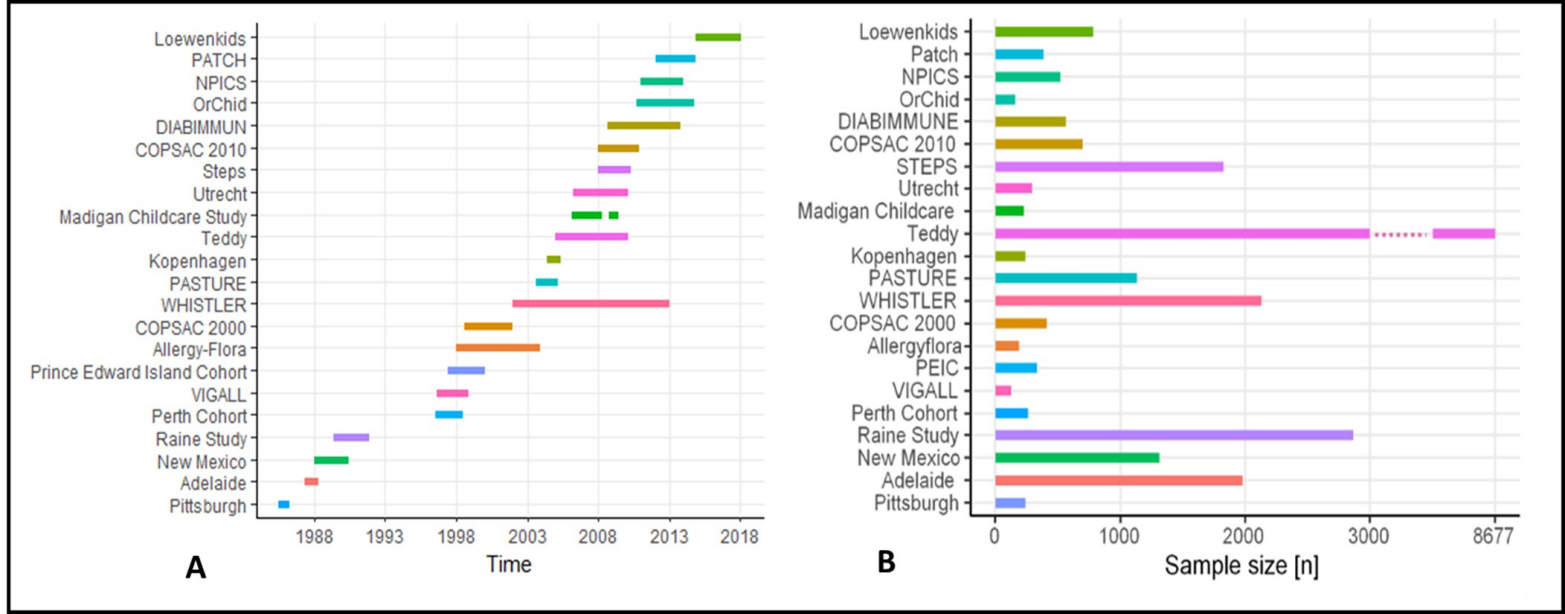
A. Time of enrollment B. Number of participants in considered studies.

The number of participants ranged from 126 in the VIGALL- Study [34] to 8677 in the Teddy-Study [35] (Fig 2A). The most recent study is the LoewenKIDS study from Germany which recruited participants between 2014 and 2016, while the oldest study is a cohort study from Pittsburgh, which enrolled subjects from July 1985 to April 1986 (Fig 2B).

Recruitment period had a median of 30 months and ranged between 9 and 133 months. Five studies are still collecting data at the time of writing, while 17 studies are completed.

### Symptom diary and follow up

In most cases, symptom diary entries were only noted when study participants showed symptoms, in others, participants were asked to enter all days including those without symptoms. The response rate of symptom diary data was reported rarely. Six years was the longest period for which symptom recording by diary was planned, while the shortest was ten months (Table 2). Seven studies collected data by symptom diary only for the first year of life or less, seven studies for the first two years, three for three years or less, two for six years, and the other studies for five years, 21 months and 18 months.

Besides using symptom diaries, thirteen studies used questionnaires to conduct the follow-up of their participants. The mode of follow-up by questionnaires ranged between monthly assessments to a follow-up interval of 17 years, but the majority of birth cohorts sent out questionnaires very frequently in the first two years after birth. In addition to questionnaires, telephone calls, interviews, email contact, home visits, and clinical examinations were used to contact the participants and collect data. Most studies used phone calls and interviews to conduct follow-ups. Nine studies carried out home visits in order to collect data, to remind participants, or to conduct routine visits during the first respiratory infection or in cases where subjects could not come to the study center. Clinical visits or medical examinations took place in 18 studies, which were usually part of a fixed follow-up-appointment. A smaller proportion of these studies encouraged their participants to visit a health center when symptoms of respiratory infections occurred.

Almost all studies analyzed individual symptoms, such as dry or wet cough, wheezing, runny or stuffy nose, sore throat, fever, chills, loss of appetite and attachment. From these symptoms, most studies derived acute respiratory infections (ARI) as outcome variables. Lower and upper respiratory tract infection (LRTI, URTI) were reported in thirteen of the studies listed. Some studies considered only individual diseases such as pneumonia, acute otitis media, or rhinitis.

We also considered the drop-out rates of the identified studies. However, not all studies reported a clear rate and those that did are very heterogeneous and not very comparable.

After six months, the New Mexico cohort recorded a drop-out rate of 12.5% [36]. The identified drop-out rates in the first year of life ranged from 10.3% in the Perth Cohort [6] and the Copenhagen Cohort [37] to 47% in the Adelaide Cohort [38]. The NPICS Cohort study was also in the lower third with 14.8% within at the first year [39]. In contrast, the STEPS study [40] reported a drop-out rate of 6% after 13 months. Three of the identified studies reported a drop-out rate of 12% after 2 years in the LoewenKIDS cohort (personal request), 32.5% in the Vigall cohort [34] and 58% in the Adelaide cohort. The PEIC cohort [41] reported a drop-out rate of 20.5% after three years. The data of all other studies considered here could not be clearly and comparably identified.

### Biosamples

Eighteen studies collected biosamples from participants during follow up periods.

#### Blood samples

The collection of blood from children was part of the protocol in thirteen studies (Table 3). Eight of these studies took additional blood samples, including umbilical cord blood (n = 8) and maternal blood (n = 7). One study collected umbilical cord blood samples only.

**Table 3:** Biological samples collected in the birth cohorts studies.

COHORT -NAME	CHILD BLOOD	ASYMPTOMATIC NASAL SWAB	SYMPTOMATIC NASAL SWAB	ASYMPTOMATIC STOOL SAMPLE	MATERNAL BLOOD	CORD BLOOD	OTHER SAMPLES
**Raine**	age of 5, 8, 14, 17, 18, 20, 22 yrs	-	-	-	18 wks, 34 wks before birth	yes	placental-, semen-, urine-, saliva sample
**VIGALL-Study**	-	-	if symptoms occur	-	-	-	-
**Perth-Cohort**	5 yrs	one in winter and a second in summer	onset of respiratory symptoms	-	-	-	-
**Allergyflora**	age of 18 m	-	-	wk 1, 2, 4, and at 6, 8, 12, 18 m, part born after 07/2000 additionally at age of 36 m	-	-	-
**COPSAC 2000**	6 m, 18 m, 1, 4, 6 yrs	during infancy	1m, 1y and if symptoms occur until 3 yrs	1w, 1, 12, 18 yrs	ad-hoc but at least 2 yrs after birth	yes	hair-, urine-, breast-milk-, saliva-sample
**COPSAC 2010**	6, 18 m, 6,8,10 yrs	1 w, 1 m, 3 m	if symptoms occur	1w, 1m, yl until 6 yrs, then at the age of 8, 10 yrs	24th wk of pregnancy	yes	hair-, urine-, breast-milk-, saliva-, skin-, dried blood sample
**PASTURE**	1, 4, 6, 10,5 y	-	-	-	at birth, at age of 1 year	yes	breast milk (2 m)
**Kopenhagen**	age 5 days, 12 m	at every home visit (mtly)	-	-	at enrolment	-	-
**Madigan Childcare Study**	-	a sample at time of enrolment	at symptom onset and wkly thereafter until asymptomatic	study 2008–2009 wkly asymptomatic and symptomatic samples	-	-	-
**TEDDY Study**	every 3 m until to the end	at 9 m of age and at each visit thereafter	-	mtly until 48 m of age, then every three m until 10 yrs, then every six m until 08/2018.	gestation/ at birth	yes	toe nail clipping, urine, saliva sample
**Utrecht-Cohort**	age of 1 m	-	at every respiratory episode	-	-	-	-
**STEPS**	1, 2 and 3 yrs	2, 13 and 24 m	during respiratory infection	at the age of one year	yes	yes	breastmilk
**WHISTLER**	-	the start of every m (subgroup)	second day of a wheezing episode (only subgroup)	-	-	-	buccal sample at birth
**DIABIMMUNE**	3, 6, 12, 18, 24, 36m	during each visit of study clinic	-	every m starting at age of 1 m	-	yes	-
**NPICS**	yl for children > 6 m	-	during visits at health centre with symptoms	-	-	-	-
**OrChid**	-	at birth and wkly	-	at birth and once a week	-	yes	
**PATCH**	-	1, 2, 4, 6, 12 m	during acute wheezy episodes	-	-	-	-
**LoewenKIDS**	age of 1 y and 2 yrs in subcohort	once per year (age 0–6 yrs; complete cohort) four times per year (age 0–2; subcohort)	if respiratory symptoms occur	once per year (age 0–6 years; complete cohort) four times per year (age 0–2; subcohort) additionally symptomatic sample if symptoms occur	-	yes	buccal sample at the age of 1 y
**wk**	**-**	**Week**	**yrs**	**years**	**data not reported**		
**wks**		**wks**	**y**	**year**			
**wkly**		**weekly**	**yl**	**yearly**			
**m**		**months**	**Mtly part**	**Monthly participant**			

#### Nasal swabs

A total of fifteen studies collected nasal swabs in different frequencies. In eight of fifteen studies, nasal swabs were taken when the child was free of respiratory infection symptoms and every time symptoms occurred, whereas in three of the fifteen studies, only asymptomatic swabs were taken.

#### Stool samples

Nine studies collected stool samples when children were free of gastrointestinal symptoms. Of these, one study additionally collected stool samples in symptomatic subjects.

#### Other biomaterials

In eight studies, genetic swabs (n = 6), urine samples (n = 4), placental sample (n = 1), toe nail clipping (n = 1), hair sample (n = 2), dried blood (n = 1), skin sample (n = 1), semen (n = 1), and samples of breastmilk (n = 4) were collected.

### Exposures and potential risk factors for respiratory infections

In most studies, known exposures and risk factors such as environmental exposures in households and outside of households, information on social contacts, socioeconomic factors, birth mode, breastfeeding, presence of siblings, animal contact, daycare-attendance, vaccination, nutrition, stress, and information about the family history were part of the collected data (Table 4). Seven studies assessed all mentioned risk factors.

**Table 4:** Recorded variables for exposures/ risk factors for respiratory infections.

COHORT NAME	RISK FACTORS												
	ENVIRONMENTAL	HOUSEHOLD EXP.	SOCIAL RELATION SHIP	SOCIOOE CONOMIC STATUS	BIRTH MODE	BREAST FEEDING	SIBLINGS	ANIMAL CONTACT	DAY CARE ATTENDANCE	VACCI NATION	NUTRITION	STRESS	FAMILY HISTORY
**Pittsburgh**	**-**	**-**	**-**	**+**	**-**	**-**	**+**	**-**	**+**	**-**	**-**	**-**	**+**
**Adelaide-Cohort**	**+**	**-**	**+**	**-**	**+**	**+**	**+**	**-**	**+**	**-**	**-**	**-**	**+**
**Raine Study**	**+**	**+**	**+**	**+**	**+**	**+**	**+**	**+**	**+**	**+**	**+**	**+**	**+**
**New Mexico**	**+**	**+**	**+**	**+**	**+**	**+**	**+**	**-**	**+**	**-**	**-**	**-**	**+**
**VIGALL**	**-**	**+**	**+**	**+**	**+**	**+**	**+**	**+**	**+**	**-**	**+**	**-**	**+**
**Perth-Cohort**	**+**	**+**	**+**	**-**	**-**	**+**	**+**	**+**	**+**	**-**	**-**	**-**	**+**
**PEIC**	**+**	**+**	**+**	**+**	**-**	**+**	**-**	**+**	**+**	**-**	**-**	**-**	**+**
**Allergyflora**	**+**	**+**	**+**	**+**	**+**	**+**	**+**	**+**	**+**	**-**	**+**	**-**	**+**
**COPSAC 2000**	**+**	**+**	**+**	**+**	**+**	**+**	**+**	**+**	**+**	**+**	**+**	**+**	**+**
**COPSAC 2010**	**+**	**+**	**+**	**+**	**+**	**+**	**+**	**+**	**+**	**+**	**+**	**+**	**+**
**PASTURE**	**+**	**+**	**+**	**+**	**+**	**+**	**+**	**+**	**+**	**+**	**+**	**-**	**+**
**Kopenhagen-Cohort**	**+**	**+**	**+**	**+**	**+**	**+**	**+**	**+**	**+**	**+**	**+**	**-**	**+**
**Madigan Childcare Study**	**+**	**-**	**+**	**+**	**+**	**-**	**+**	**+**	**+**	**-**	**-**	**-**	**+**
**TEDDY**	**+**	**+**	**+**	**+**	**+**	**+**	**+**	**+**	**+**	**+**	**+**	**+**	**+**
**Utrecht-Cohort**	**-**	**+**	**+**	**+**	**+**	**+**	**+**	**+**	**+**	**-**	**+**	**-**	**+**
**STEPS**	**+**	**+**	**+**	**+**	**+**	**+**	**+**	**+**	**+**	**+**	**+**	**+**	**+**
**WHISTLER**	**+**	**+**	**+**	**+**	**+**	**+**	**+**	**+**	**+**	**-**	**+**	**-**	**+**
**DIABIMMUNE**	**+**	**+**	**-**	**-**	**+**	**+**	**+**	**+**	**+**	**+**	**+**	**-**	**+**
**NPICS**	**+**	**+**	**+**	**+**	**+**	**+**	**+**	**+**	**+**	**+**	**+**	**+**	**+**
**OrChid**	**+**	**+**	**+**	**+**	**+**	**+**	**+**	**+**	**+**	**+**	**+**	**-**	**+**
**PATCH**	**+**	**+**	**+**	**+**	**+**	**+**	**+**	**+**	**+**	**+**	**+**	**-**	**+**
**LoewenKIDS**	**+**	**+**	**+**	**+**	**+**	**+**	**+**	**+**	**+**	**+**	**+**	**+**	**+**

+ yes

- data not recorded

## Discussion

In this scoping review, we identified 22 birth cohort studies using a symptom diary to collect symptoms of respiratory infections beginning within the first four months after birth. These studies varied in terms of number of participants, duration of data collection, follow-up times, as well as in respiratory outcomes, collected biosamples and assessed environmental exposures.

The number of detected birth cohort studies employing diaries is small compared to the overall number of birth cohort studies that were established on bronchial asthma, allergies, and respiratory infections over the past 35 years. Some excluded studies used symptom diaries after the first year of life and the majority of the excluded studies used other approaches like retrospective questionnaires, interviews and clinical visits to estimate the burden of respiratory diseases [17–23].

While episodes of respiratory infections can be recalled fairly well over a period of two months [88–90], it is unlikely that daily symptom evolution can be accurately recalled without prospective daily collection. Therefore, it is unlikely that the sequence of symptoms, especially if they were frequent and less severe, can be recalled with any degree of accuracy using retrospective records, thereby introducing recall bias into these studies [91]. Preventing this is particularly important for transient symptoms of childhood infections, especially when we are interested in the prospective evolution of symptoms. This includes duration, intensity, and whether only one or multiple symptoms occurred [52]. Therefore, symptom diaries have been used for many decades. They produce more valid data, i.e. higher reporting and incidence rates thereby mitigating recall bias [13, 91, 92].

### Causes of low symptom diary use in birth cohort studies

An overall aim of this review was to map birth cohorts that use(d) symptom diaries to identify respiratory diseases. Of the numerous birth cohort studies identified, relatively few really used a symptom diary to identify respiratory illness. Substantial administrative study efforts, the effort involved in data analysis, but also possible upcoming problems due to drop-out rates, could be possible barriers from the researchers’ point of view. In addition, filling out a daily symptom diary is a considerable burden for participants. Some studies show that, although the respondents themselves report good compliance, data collection protocols are often not followed, and a large number of missing records might occur [93, 94]. On the other hand, one reason for the rather infrequent use may be the risk of "hoarding", which is known as a problem where participants enter data into their diary retrospectively [93]. Unfortunately, hoarding is almost impossible to detect unless the time of data entry is recorded electronically. Weariness can also lead to a decline in diary completion rates over time [95]. It is therefore important to have a well-staffed and well-trained study team that can maintain good contact with the study participants to avoid missing data and drop-out.

This review shows that especially immediately after birth, when infants show a high susceptibility to respiratory illness, few studies use symptom diaries to record respiratory symptoms[96]. Because the immune system undergoes crucial developmental maturation during this time, detailed recording of all potential exposures, including infections, is crucial in understanding the development of the immune system and other outcomes. Most of the studies identified here were conducted in Europe, North America, and Australia, which may limit the generalizability of results and thus require more birth cohorts from other regions of the world.

Some studies retrieved either ARI, URTI or LRTI as respiratory outcomes from the symptom diary data, yet there is no consensus how to use the collected data in harmonized manner. This also makes it difficult to compare respiratory outcomes in this ScR. Additionally, most studies collected a broad range of environmental exposures, but only a few collected a broad range of biosamples. Nevertheless, biosamples are important because, they complement associations and research questions. For example, nasal swabs can be used to identify pathogens responsible for the development of ARI and to study them in relation to a specific combination of symptoms. Analysis of other biosamples, such as blood samples, can help to understand the immune system’s response to the development of ARI.

### Strengths and limitations

To our knowledge, this scoping review is the first comprehensive attempt to summarize, map and compare birth cohort studies with symptom diary information for respiratory symptoms beginning from birth. The strength of our review is the comprehensive search of the literature. Our rather specific search strategy was comprehensively extended by a systematic search in four databases, an additional extensive search in registers and a very elaborate search in reference lists of the identified publications from all four databases. We assume that due to this extended search strategy the probability of having overlooked a birth cohort study with the use of symptom diaries from birth is low.

We also conducted additional searches of the gray literature to avoid omitting cohorts that had recently started. Finally, to ensure the accuracy of the extracted data, we contacted one or two authors of all included birth cohorts to confirm the extracted information or clarify, if questions remained open. Our study also had several limitations. It is possible that we failed to identify some cohort studies applying symptom diaries due to no uniform wording for symptom diaries. Not all birth cohort studies offer detailed cohort profiles, and information had to be collected in different publications. In addition, it may be possible that a birth cohort study could not be identified because it is neither registered in one of the web-based registers nor published in a way that met our search criteria. We hope that the broad search allowed to minimize these problems.

## Conclusions

We have been able to provide a comprehensive review with all birth cohort studies that are suitable for providing a holistic understanding of the association of ARI as exposure with potential long-term sequelae in a life course perspective. We found 22 birth cohort studies that use(d) symptom diaries for respiratory infections. While, symptom diaries provide a powerful tool for prospective data collection, their long-term application is very challenging, so this is why it is not often applied. Our review shows that is possible and was done in several studies. Many questions related to the role on infections in the development of the immune system require information on symptom evolution and infection history over time. When combined with biosamples, this detailed information is very valuable. This overview helps to establish collaborations between researches in order to investigate the pattern, timing and sequence of respiratory infections and their association with the developing immune system and other exposures in a life-course perspective.

## Supporting information

S1 Checklist Preferred reporting items for systematic reviews and meta-analyses extension for scoping reviews (PRISMA-ScR) checklist. (DOCX)

## Abbreviations

ARI: Acute respiratory infection
ScR: Scoping Review
AUS: Australia
CAN: Canada
SWE: Sweden
CHE: Switzerland
FRA: France
AUT: Austria
EUR: Europe
USA: United States of America
DK: Denmark
NLD: Netherlands
FIN: Finland
NIC: Nicaragua
AUS: Australia
TWN: Taiwan
DEU: Germany
RUS: Russia
EST: Estonia
